# Patients’ views on an education booklet following spinal surgery

**DOI:** 10.1007/s00586-012-2242-y

**Published:** 2012-03-02

**Authors:** A. H. McGregor, A. Henley, T. P. Morris, C. J. Doré

**Affiliations:** 1 Surgery and Cancer, Faculty of Medicine, Imperial College London, Charing Cross Hospital Campus, London, W6 8RP UK; 2 MRC Clinical Trials Unit, 222 Euston Road, London, NW1 2DA UK

**Keywords:** Evidence-based booklet, Education, Spinal surgery, Acceptability, Surgical journey

## Abstract

**Purpose:**

This study evaluated an evidence-based education booklet developed for patients undergoing spinal surgery which was used as a treatment intervention in a multi-centre, factorial, randomised controlled trial (FASTER: Function after spinal treatment, exercise and rehabilitation) investigating the post-operative management of spinal surgery patients. This study sought to determine the acceptability and content of the booklet to patients.

**Methods:**

Patients receiving the educational booklet before discharge from hospital as part of the FASTER study were asked to complete an evaluation, which rated the booklet “Your Back Operation” with regard to content, information, usability, etc. using forced and open questions. This assessment was conducted at the same time as the initial 6-week post-operative review performed as part of the larger study.

**Results:**

Therefore, 97% of the 117 trial participants who returned their 6-week evaluation and randomised to receive a booklet returned their questionnaire. The booklet was highly rated receiving an overall rating of 7 or more out of 10 from 101/111 (91%), and high ratings for content, readability and information. The booklet’s key messages were clear to the majority of patients; however, many patients highlighted deficiencies with respect to content particularly in relation to wound care and exercise.

**Conclusions:**

Patients valued the booklet and rated its content highly. Many suggested that the booklet be developed further and there was a clear desire for specific exercises to be included even though there is no evidence to support specific exercise prescription.

## Introduction

Patient education has a long history in medicine, regaining prominence in the 1960s with the emergence of health promotion campaigns [26]. Patient education is frequently used as a component or adjunct to the management of a medical condition or disease process [5]. It has been described as a planned, organised learning experience designed to facilitate voluntary adoption of behaviours or beliefs conducive to health through influencing the patient’s knowledge and health behaviour [4, 6, 9].

Traditionally patient education has focussed on providing information and technical skills, however, there is a move towards self-management through directed education, which facilitates patients taking an active role in identifying their problems, and provides techniques and skills to help them make decisions and take appropriate actions as they encounter changes in their disease or circumstances [8]. In this way patients are more likely to become empowered, possibly through improved self-efficacy [17] and as a result tend to take greater responsibility for their own condition [3, 8]. Indeed, there is growing evidence that self-management has a greater impact on the disease process than didactic education [7].

Modern technology now provides a wealth of formats for the transfer of educative information; however, at present not all of these technologies are readily available to patients. Key formats are either educational materials such as booklets and leaflets, educational classes, or oral advice. Enger et al. [12] reviewed the role of patient education for low back pain and found limited differences between the various approaches to patient education. They made no recommendations on the form, content, intensity or frequency of the information but did consider that information could be as effective as non-educational interventions particularly in the management of acute back pain. Less is known with regard to surgical interventions.

In the surgical environment, there is a growing body of evidence indicating the need and importance of discharge information [1, 10, 11, 14]. Inadequate discharge information has contributed to poor patient satisfaction, increased stress and anxiety, inability to cope, increased consultation and admission rates, and poor treatment adherence [11]. This is particularly pertinent for spinal surgery, where a recent survey evaluating post-operative management revealed wide variation in recommendations for activity and return to work following surgery [19]. Thus whilst individual surgeons were certain of their practice, the overall variation identified in post-operative management demonstrates uncertainty amongst the profession. This in part may contribute to the mixed levels of patient satisfaction noted following spinal surgery [2, 23, 32], and the variable outcomes [15, 22, 27, 29, 32] and quality of life changes reported [16, 28]. Building on the success of “The Back Book” a patient orientated evidence-based booklet for back pain [25] and following a review of the literature [18], an evidence-based patient booklet for patients undergoing discectomy and un-instrumented spinal fusion—“Your Back Operation”—was developed and published [30]. Whilst both patients and surgeons welcomed the booklet a full evaluation was not performed and subsequently the booklet was factored into a randomised controlled trial of rehabilitation strategies for post-operative management after lumbar surgery [20]. The current study focusses on a sub-protocol of this larger study to evaluate the booklet from the patient’s perspective in terms of length, structure, style and content.

## Methods

The data collected in this study were part of a multi-centre, factorial, randomised controlled trial comparing the effectiveness of a rehabilitation programme and an education booklet for the post-operative management of patients undergoing discectomy or lateral nerve root decompression, each compared with “usual care”, the details of which have been previously reported [21, 24]. Regional and local ethical approval was obtained and patients recruited into the study underwent randomisation stratified by surgeon and procedure using permuted blocks. The study created 4 sub-groups: rehabilitation-only, booklet-only, rehabilitation-plus-booklet, and usual-care-only. Standard outcomes measures (function, pain, cost, anxiety, distress) were obtained by patient completed questionnaire pre-operatively and then at 6 weeks, 3, 6 and 9 months and 1 year post-operatively [20]. All surgeons were fully briefed on the study and endorsed and supported the trial interventions. This current paper concerns the booklet only and the booklet plus rehabilitation group from this larger trial.

### Study population

Patients were recruited by the trial coordinator from the surgical waiting lists of the contributing surgeons (20 in total; 8 orthopaedic and 12 neurosurgical) at the seven different hospital sites and written informed consent obtained. Eligible patients included those awaiting spinal surgery with either (a) signs, symptoms and radiological evidence of lateral nerve root compression, that is, patients presenting with radicular pain with an associated neurological deficit or with neurogenic claudication (pain in the buttock, thigh or leg that improves with rest), or (b) lumbar disc prolapse, that is, patients with root symptoms and signs and MRI confirmation of lumbar disc herniation. All participants underwent spinal surgery according to their surgeon’s routine practice.

Patients presenting with any of the following were excluded from participation: any condition where either the intervention or the rehabilitation may have an adverse effect on the individual; previous spinal surgery; spinal surgery where a fusion procedure was planned due to the unknown hazards of the activity programme for this type of surgery; pregnant women; inadequate ability to complete the trial assessment forms; unable to attend or unsuitable for rehabilitation classes.

### Post-operative trial interventions

#### Rehabilitation programme

Patients randomised to the rehabilitation arms of the trial were invited to 12 1 h classes run by an experienced physiotherapist starting 6–8 weeks following their surgery. The classes were standardised to a set protocol with clear exercises, and progression and included general aerobic fitness work; stretching; stability exercises; strengthening and endurance training for the back, abdominal and leg muscles; ergonomic training; advice on lifting and setting targets; and self-motivation along with an open group discussion at the end of each class where problems and concerns could be discussed with the therapist.

#### Educational booklet

Patients randomised to the booklet arms of the trial received a copy of “*Your Back Operation*” [30] from the trial coordinator on discharge from hospital. The design and content of the booklet have been previously reported [18].

#### Usual care

Patients randomised to the usual care control group were managed according to the relevant surgeon’s usual practice. For the majority of participating surgeons this was limited to brief advice to be active and a follow-up review within 6–12 weeks.

### Booklet evaluation

At the 6-week post-operative review, those patients randomised to receive the educational booklet were also sent a questionnaire to evaluate the booklet they had received. The design of the questionnaire has been previously reported [18] and comprised two parts: a series of 11 forced-choice questions on readability, style, information level, believability, length, content and helpfulness; and open questions about the most important messages they took from the booklet, anything they did not like or understand, if they had any concerns that were not covered, if they thought the booklet would change what they did after surgery, and finally their overall rating of the booklet on a scale from 1 to 10.

### Statistical methods

Mann–Whitney *U* tests were performed to compare the booklet evaluation scores between the booklet only and booklet plus rehabilitation groups, and between the two types of surgery to investigate whether receiving the booklet influenced the acceptability of rehabilitation. These sub-analyses were performed to explore any potential gaps in the booklet identified by patients not having the rehabilitation classes and in response to differences in outcome observed in the main trial outcome paper [21].

## Results

Of the 161 patients randomised to receive the booklet, 91 (57%) were allocated to the booklet and rehabilitation group and 70 (43%) to the booklet only group. At the 6-week post-operative review, one subject had withdrawn from the booklet and rehabilitation group, 1 was lost to follow-up and 16 missed this review stage. With respect to the booklet only group, 2 withdrew, 4 were lost to follow-up and 20 missed this review stage. Of the remaining 117 who responded to this review across the 2 groups, 114 subjects returned their booklet evaluation (45 in the booklet only group and 69 in the booklet and rehabilitation group).

Considering the respondents as a whole, the majority felt the booklet was easy to follow and found the content interesting (75%), stating that they learned new and helpful facts (78%), see Table 1. Some felt they learned nothing new from the booklet (20%) and others felt it was simply not helpful (3%); 99% found the booklet easy to follow, while 94% would recommend it to a friend or family member.

**Table 1:** Summary of responses to the booklet evaluation by group and procedure

	Booklet only	Booklet plus rehabilitation	Decompression surgery (booklet only/booklet plus rehabilitation)	Discectomy surgery (booklet only/booklet plus rehabilitation)
Content	*n* = 45	*n* = 68	*n* = 62	*n* = 51
Interesting	33 (73%)	52 (76%)	47 (76%)	38 (75%)
OK	12 (27%)	14 (21%)	14 (23%)	12 (24%)
Boring	0 (0%)	2 (3%)	1 (2%)	1 (2%)
Readability	*n* = 45	*n* = 69	*n* = 63	*n* = 51
Very easy	33 (73%)	51 (74%)	45 (71%)	39 (76%)
Quite easy	7 (16%)	11 (16%)	11 (17%)	7 (14%)
Just right	5 (11%)	7 (10%)	7 (11%)	5 (10%)
Informative	*n* = 43	*n* = 69	*n* = 62	*n* = 50
Yes	36 (84%)	51 (74%)	49 (79%)	38 (76%)
I knew most of it anyway	6 (14%)	16 (23%)	11 (18%)	11 (22%)
No	1 (2%)	2 (3%)	2 (3%)	1 (2%)
Length	*n* = 44	*n* = 69	*n* = 63	*n* = 50
Too long	1 (2%)	3 (4%)	3 (5%)	1 (2%)
About right	41 (93%)	60 (87%)	57 (90%)	44 (88%)
Too short	2 (5%)	6 (9%)	3 (5%)	5 (10%)
Easy to follow	*n* = 42	*n* = 68	*n* = 61	*n* = 49
Yes	42 (100%)	67 (99%)	60 (98%)	49 (100%)
Recommend this to a friend	*n* = 44	*n* = 69	*n* = 63	*n* = 50
Yes	42 (95%)	64 (93%)	58 (92%)	48 (96%)
How often did you use	*n* = 43	*n* = 69	*n* = 62	*n* = 50
Never	9 (21%)	12 (17%)	11 (18%)	10 (20%)
Occasionally	32 (74%)	41 (59%)	39 (63%)	34 (68%)
Numerous times	2 (5%)	16 (23%)*	12 (19%)	6 (12%)
Was everything covered?	*n* = 40	*n* = 62	*n* = 57	*n* = 45
Yes	26 (65%)	44 (71%)	38 (67%)	32 (71%)
Overall rating out of 10	*n* = 43	*n* = 68	*n* = 61	*n* = 50
Median, range, % scoring 7 or above	8, 5–10, 88	8.5, 1–10, 93	9, 1–10, 90	8, 5–10, 92

* Significance *p* < 0.05

Whilst the majority (69%) were content with the booklet content, 31% of responders indicated that the booklet had deficiencies. This was mainly in relation to exercise, with nearly half of these respondents wanting greater detail with respect to type, duration, frequency and intensity of exercises. Other issues pertained to the need for more information on pain control, driving, and medical procedural information such as wound care and infection management/prevention. Of the respondents, 31% also requested for more practical tips to follow. When asked to rate the overall performance of the booklet of out 10, the median score was 8 and the range was 1–10, although 91% rated it 7 or above. Many patients read the booklet more than once, with 65% referring to it occasionally and 16% frequently referring to it during their recovery. Responses were similar for the two surgical procedures and for the two randomised groups (Table 1), although the booklet plus rehabilitation group used the booklet significantly more often (*p* = 0.03).

When subjects were asked to identify the three most important messages from the booklet a surprising range of responses were received. The message to be active and to exercise was clearly identified by the majority of respondents and a substantial proportion of respondents noted the value of staying positive. Other common responses related to understanding the symptoms and aspects of the surgery performed. Some simply found that the booklet gave them hope and re-assurance. However, several participants failed to answer this section, with others simply stating that they enjoyed reading it and found all the information and messages important, particularly the knowledge that they were not the only person to experience this problem. Of concern, two respondents felt the booklet conveyed the need to be cautious or disciplined with exercise and activities. Others simply praised the practical tips, and the need to realise that one has good days and bad days and not to be discouraged by the bad days. One respondent was particularly negative stating that none of the information in the booklet would get them better.

Participants were invited to provide some free comments on the booklet and its content. A recurrent request was to have received the booklet pre-operatively, however, to reduce the risk of group contamination the trial protocol dictated that the booklet was only received on discharge. Again, requests were made for greater detail, more specifics on exercise and rehabilitation and for links to other useful sources of material. One participant commented that whilst they agreed with the need to exercise they lacked the discipline to do this, and another stated that the booklet made them more anxious as they were not recovering as well as the booklet implied. Many positive comments were also received including that the booklet was excellent and should go to all NHS patients, that it was helpful, reassuring and confidence building and that it helped them to understand a complex problem in a simple way.

## Discussion

Over recent years, there has been a growing recognition of the need for educational materials for patients [4, 5], and increasingly patients are becoming active healthcare partners [13]. This education should not simply be considered as providing facts for patients, but should be seen as a “learning experience” that facilitates the development of new behaviours, skills, and beliefs conducive to health [6, 9], and with this the ability to foster concepts of self-management [8]. The importance of educational material is reinforced by a recent audit commission finding that patients do not receive sufficient information to address their needs [5], and failure to meet a patient’s information needs has been identified as a prime source of dissatisfaction with the healthcare system [31].

From the evaluations received it would appear that “*Your Back Operation*” was well received and liked with most of the patients rating it very highly and happy to recommend it to a friend. This is in line with the early pilot performed on the booklet as part of its development [18]. We had not anticipated that the group receiving both rehabilitation and the booklet would refer to the booklet more often and in fact would have expect the reverse, this however may be a spurious finding and related to the booklet being received on discharge and rehabilitation 6 weeks later. While reading rates were high in both groups, how this would translate to clinical practice is unclear: it is possible that the review questionnaire prompted patients to re-read or return to their booklets. More importantly it was reassuring that some of the key messages got through to patients particularly in regard to being active and positive. However, this was not the case for all with many focussing on the details of the operation and understanding what was wrong with them.

The booklet was designed to be evidence based, but due to the lack of published evidence on specific exercises or stretches in the literature in regard to the post-operative management of back pain and indeed chronic back pain a decision was made to make the exercise advice very open and focussed on simply getting moving and being active. In the evaluations of the booklet however, it became clear that patients wanted specific exercises and stretches, favouring a clear regime rather than simple open advice. This creates a dilemma in terms of what to include in such a regime, and is clearly an area for future work since it would have to be safe, clear and appropriate for a range of patient’s abilities and fitness levels. However, it is encouraging as it does support the notion that patients want to be active partners in their recovery [13]. There are also parallels with a study by Anelise-Santo et al. [1] looking at the education of parents whose child was having spinal fusion surgery. They noted that whilst the parents received information on aspects of what to do they were not provided with the tools to achieve these targets, i.e. there was no opportunity for skill development. Whilst our patients knew they should exercise we may not have provided them with the skills or indeed confidence to implement this advice.

Few other studies have looked at patient education following spinal surgery however Engers et al. [12] performed a Cochrane review of educational approaches used in the management of back pain including booklets, books, video tape and simple oral communication. The results were mixed in that although education was highlighted as important and effective, the appropriate form, content, intensity, and frequency were unclear [12]. From this study, it would appear that the booklet was liked and well received by patients and with some revisions it could be a useful adjunct to patients’ recovery from surgery, however, we need to more fully understand its impact on outcome [21]. Key areas for revision for the booklet should include the provision of clear recovery milestones including time to return to driving etc., clear discharge information on wound care and dressing changes, more information on pain control, and a clearer set of exercises and stretches for the patient to do.

In conclusion, the booklet was welcomed by patients and they valued the information and support it contained. However, clearly not all aspects of care were covered, and in some instances the messages to return to activity and an active lifestyle were not understood. If revised in the future, many of the negative aspects identified by this study need to be addressed and clearer strategies to change behaviours and lifestyles need to be integrated into the messages such that patients are aware that they need to adopt an active role and enhance and build upon their pre-operative status.

## FASTER team

**Trial Steering Committee**

- Professor Jeremy Fairbank
- Mrs Caroline Doré
- Lord ER Oxburgh
- Professor Jennifer Klaber Moffett
- Mr Steve Eisenstein
- Mr Tim Morris
- Professor Alison McGregor
- Professor Stephen Morris
- Professor Konrad Jamrozik
- Trial Coordinator (see below)

**Trial Management Team**

- Principle investigator: Alison McGregor
- Trial Coordinators: Ania Henley, Jack Kerr, Dr Julia Toschke
- Physiotherapy Coordinators: Carla Ashford, Janet Deane, Carol Waugh

**Data Monitoring and Ethics Committee**

- Professor Sarah Hewlett
- Dr Simon Bowman
- Dr Martyn Lewis

**Participating Hospitals**

- Charing Cross Hospital
- Guy’s and St Thomas’ Hospital
- Heatherwood Hospital
- Ravenscourt Park Hospital
- Royal Free Hospital
- St Mary’s Hospital
- Wexham Park Hospital

**Contributing Surgeons**

- Mr M Akmal
- Mr N Anjarwalla
- Mr R Bradford
- Mr N Dorward
- Mr T Ember
- Mr D Fahy
- Professor S Hughes
- Mr J Johnson
- Mr K Lam
- Mr J Lucas
- Mr N Mendoza
- Ms M Murphy
- Mr D Nandi
- Mr J O’Dowd
- Mr K O’Neill
- Mr D Petersen
- Mr C Shieff
- Mr L Thorne
- Mr C Ulbricht
- Professor J van Dellen

**Trial Physiotherapists**

- Katie De Albuquerque
- Nathan Allwork
- Hayley Boyle
- Ann Bryan
- Rachel Freeman
- Jenny Heal
- Annys Hawkins
- Catherine Heathcote
- Frances Honniball
- Gary Jones
- Biljana Kennaway
- Gemma Kettle
- Anna Kuppuswamy
- Claire Marshall
- Ann McCarthy
- Hannah Mundy
- Sandra Noonan
- Sara Parker
- Camilla Pleydell-Bouverie
- Rebecca Portwood
- Amy Powel
- Adam Royffe
- Victoria Salmon
- Laura Segar
- Alison Sentence
- Tom Stenning
- Hillary Thompson
- Lorraine Tomeldan
- Amanda Wall
